# A new photodetector structure based on graphene nanomeshes: an ab initio study

**DOI:** 10.3762/bjnano.11.88

**Published:** 2020-07-15

**Authors:** Babak Sakkaki, Hassan Rasooli Saghai, Ghafar Darvish, Mehdi Khatir

**Affiliations:** 1 Department of Electrical and Computer Engineering, Science and Research Branch, Islamic Azad University, Tehran, Iran; 2 Department of Electrical Engineering, Tabriz Branch, Islamic Azad University, Tabriz, Iran

**Keywords:** absorption spectra, DFT calculations, graphene nanomesh, graphene nanoribbon, photodetectors

## Abstract

Recent experiments suggest graphene-based materials as candidates in future electronic and optoelectronic devices. In this paper, we propose to investigate new photodetectors based on graphene nanomeshes (GNMs). Density functional theory (DFT) calculations are performed to gain insight into electronic and optical characteristics of various GNM structures. To investigate the device-level properties of GNMs, their current–voltage characteristics are explored by DFT-based tight-binding (DFTB) in combination with non-equilibrium Green’s function (NEGF) methods. Band structure analysis shows that GNMs have both metallic and semiconducting properties depending on the arrangements of perforations. Also, absorption spectrum analysis indicates attractive infrared peaks for GNMs with semiconducting characteristics, making them better photodetectors than graphene nanoribbon (GNR)-based alternatives. The results suggest that GNMs can be potentially used in mid-infrared detectors with specific detectivity values that are 100-fold that of graphene-based devices and 1000-fold that of GNR-based devices. Hence, the special properties of graphene combined with the quantum feathers of the perforation makes it suitable for optical devices.

## Introduction

Graphene monolayers with honeycomb crystal structure have unique electrical and optical properties and have received a lot of attention recently among experimental and theoretical researchers. Electrical properties such as a charge mobility in the range of 10^5^ cm^2^·V^−1^·s^−1^, a minimum conductivity at the Dirac point of 4*e*^2^/π*h* (at low temperature), and remarkable optical properties such as linear dispersion of the Dirac electrons make broadband applications possible. Saturable absorption, as a consequence of Pauli blocking and non-equilibrium carriers, results in hot luminescence that cannot be attained in conventional semiconductors. Hence, these properties make it an ideal photonic and optoelectronic material [1–4].

Photodetectors are a key technology in all modern optoelectronic devices. Recently, researchers were studying the use of graphene and graphene-based materials in detector devices [5–17]. However, the gapless nature of graphene limits its applications in photodetectors considerably. In particular, photodetectors based on graphene will have a large dark current due to the conductivity of graphene even without incident photons [2].

An energy gap in the band structure of graphene can be created using quantum confinement effects via creating graphene nanoribbons (GNRs) with a width of nanometers [18–22]. Also, defects in GNRs have been one way of engineering the absorption spectrum of detectors based on these materials. We have shown that defects in GNR transistors can improve the device performance [23], but the limitation of GNRs to carrying the current and the fabrication of GNRs with precise width are challenges concerning these materials. In addition to the electrical properties of GNRs, the use of these materials for the manufacturing of optical detectors has been extensively investigated.

Another graphene semiconductor material are graphene nanomeshes (GNMs). The GNMs have been studied in devices such as transistors with GNM channels. The devices have been shown to have a higher on–off current than graphene transistors or nanoribbon transistors (approximately 100-fold). Detectors in which GNMs are used as the transmission channel have also been reported [24].

In this paper, for the first time, we study a new GNM-based photodetector using computational modeling. In order to do a complete device simulation, we initially perform ab initio DFT calculations to investigate the electronic and optical properties of the several materials used in devices channels, and then proceed with the design of the photodetector based on a suitable material. We further calculate the transport and photocurrent of the devices. To demonstrate improved performance of the proposed devices, we perform simulations on three types of devices. Graphene, GNMs and GNRs are materials that we employ as a device channel.

## Photodetector Structure Design

**Figure 1:**
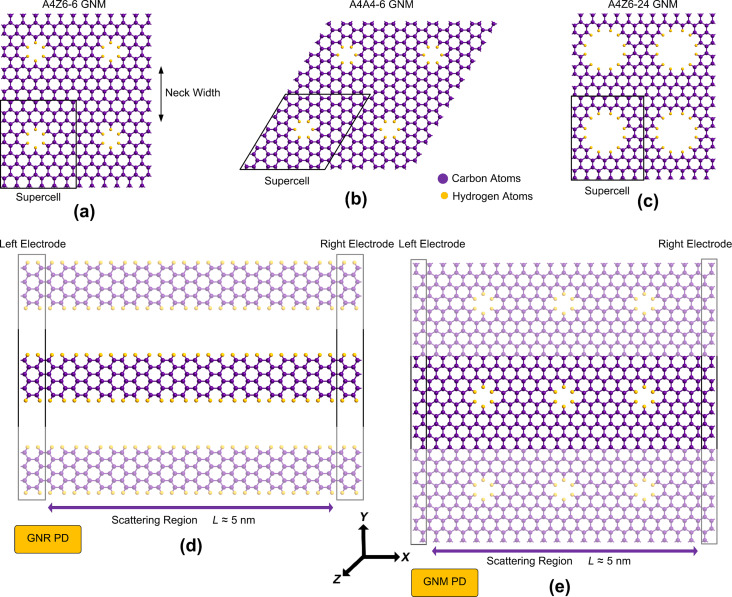
Graphene nanomesh structures. (a) A4Z6-6, (b) A4A4-6, and (c) A4Z6-24. Purple (larger) and yellow (smaller) spheres represent carbon and hydrogen atoms, respectively. Quadrilaterals with black lines represent the supercells of GNM. (d) A photodetector device made of GNRs with two source and drain contacts. The contacts are made of doped GNRs and the channel of device is periodic in the *Y* direction. (e) A photodetector device made of a GNM with two source and drain contacts. The contacts are made of graphene and the GNM channel of the device is periodic in the *Y* direction.

Figure 1a–c shows structures of A4Z6-6, A4A4-6 and A4Z6-24 GNMs with their supercells indicated by quadrilaterals. In our notation, A and Z represent the edges of the GNM supercell along the armchair and zigzag directions of the graphene lattice, respectively. That is, in A4Z6-6, A4 and Z6 represent two translational vectors the magnitudes of which are four armchair and six zigzag atomic arrangements, respectively. The figure six at the end indicates the number of carbon atoms eliminated from the supercell. As indicated in Figure 1, from the A4Z6-6 and A4A4-6 supercells six carbon atoms (one hexagon) were removed, and from the A4Z6-24 supercell 24 carbon atoms were removed. Note that the creation of these holes in the graphene lattice leaves dangling bonds on carbon atoms at the edges and increases the reactivity of these atoms. Carbon atoms located at the edge of the holes can react easier with substrate atoms. While these atoms can be passivated with hydrogen and oxygen, we chose to use hydrogen.

Figure 1d and Figure 1e show photodetector devices that are made of GNR and GNM materials, respectively. In the former, the channel is made of a large number of GNRs of the same type arranged in the *XY* plane in a parallel fashion, and the device channel in the *Y* direction is periodic. A finite number of GNRs, each with a constant length, is placed between the electrodes in *X* direction. The electrodes and the channel are made of the same GNR type. Also, we consider the electrodes have n-type doping. The left- and the right-hand edge of the electrodes are shown in the figure with black lines.

Figure 1e shows a typical GNM-based device where nanomesh channel with graphene contacts are introduced to be used in our simulations. We assumed a finite GNM length between the two contacts. The channel of the photodetector has three GNM supercells along the transport direction (*X* direction), while in the perpendicular direction the channel length is defined to be infinite.

To compare the designed devices with other photodetectors, we also design and study photodetectors based on GNRs and pristine graphene and repeat the device-level calculations. Thus, we consider three types of devices that have the same structure but use three different types of material in the channel. The length of the channel in all of these devices is about 5 nm. The electrodes on either side of the graphene and GNM devices are made of graphene. The electrodes in all three types of devices are n-type doped.

## Computational Method

In the first section of calculations, we perform DFT calculations by using ATK software with linear combination of atomic orbitals (LCAO) basis set. Generalized gradient approximation (GGA) is used for the exchange–correlation functional. A 9 × 9 *k*-point grid and a cutoff energy of 160 Ry are used to perform these calculations. All nanostructure geometries are relaxed.

To evaluate optical absorption, the susceptibility tensor is calculated using the Kubo–Greenwood formula [25–26]:

[1]
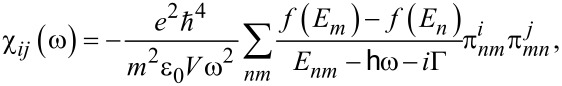


where 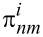 is the *i*-th component of the dipole matrix element between states *n* and *m*, Γ is the broadening, and *f* is the Fermi function. The constants *e*, ℏ*, m*, ε_0_ and *V* are the electron charge, Planck’s constant, electron mass, vacuum permittivity and system volume, respectively.

The relative dielectric constant, ε_r_, is related to the susceptibility, χ, as [26–27]:

[2] $$\varepsilon_{\text{r}}\left(\omega \right)=1+\chi \left(\omega \right).$$

The photocurrent is calculated by first-order perturbation theory in the framework of the Born approximation. In short, light–electron interaction is added to the Hamiltonian as [28–29]:

[3] $$\hat{H}={\hat{H}}_{0}+\frac{e}{m_{0}}A\cdot \hat{P},$$

where 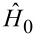 is the Hamiltonian without light–electron interaction and *e* is the electron charge, *m*_0_ is the free electron mass, 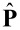 is the momentum operator, and **A** is the electromagnetic vector potential. Assuming a single-mode monochromatic light source, the first-order Born electron–photon self-energies are [28–29]:

[4] $$\Sigma_{\text{ph}}^{>}=\left[NM^{†}G_{0}^{>}\left(E^{+}\right)M+\left(N+1\right)MG_{0}^{>}\left(E^{-}\right)M^{†}\right],$$

[5] $$\Sigma_{\text{ph}}^{<}=\left[NMG_{0}^{<}\left(E^{-}\right)M^{†}+\left(N+1\right)M^{†}G_{0}^{>}\left(E^{-}\right)M\right],$$

where *E*^±^ = *E* ± ℏω. **M** is the first-order coupling matrix and *N* is the number of photons per unit of time per unit area. Thus, the Green’s functions involving the first-order electron–photon interaction are given as [28–29]:

[6]
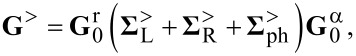


[7]
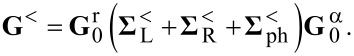


In Equations 4–7, 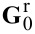, 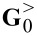 and 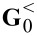 represent the non-interacting Green’s functions and α is the lowest and highest self-energies due to coupling to the electrodes. The current at the electrode α (left to right) with spin σ is calculated using [28–29]:

[8]
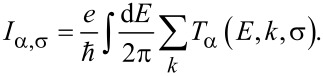


The following relation determines the effective crossing coefficients [30]:

[9] $$T_{\alpha }\left(E,k,\sigma \right)=Tr{\left\{i\Gamma_{\alpha }\left(E,k\right)\left[1-f_{\alpha }\right]G^{<}+f_{\alpha }G^{>}\right\}}_{\sigma \sigma }.$$

To investigate and compare photodetectors, we calculate figure of merits (FOMs) for these devices. FOMs such as quantum efficiency (QE), responsivity and dark-current limited detectivity (D*) are evaluated. The quantum efficiency, η, is defined as the ratio of the number of electrons in the external circuit produced by an incident photon of a given wavelength, and the basic formula for the responsivity, *R*, is given as:

[10] $$R=\eta \frac{q}{hf},$$

where *h* and *f* are the Planck constant and the frequency of incident photon, respectively. Hence, the formula denotes a direct correlation between responsivity and quantum efficiency. The dark-current limited detectivity can be defined via the following formula:

[11] $$D^{*}=R\sqrt{\frac{A}{4eJ_{\text{dark}}H},}$$

where *R*, *A*, and *H* are responsivity, length of active region, and width of the device, respectively. *J*_dark_ is the dark-current density obtained from transport analysis in the absence of light. To calculate the dark current of the proposed photodetectors at different bias voltages, we use the density-functional-based tight-binding (DFTB) method in combination with non-equilibrium Green's functions (NEGFs). As an approximate density-functional method, DFTB holds nearly the same accuracy, but at much lower computational cost, allowing for the investigation of the electronic structure of large systems, which cannot be obtained from conventional ab initio methods.

## Results and Discussion

### Electrical and optical properties

We first calculate the electronic properties of the GNR and GNM structures using ab initio calculations and then obtain the absorption spectrum of these materials using optical analysis. Finally, by calculating the photocurrent of the detectors based on these materials, we discuss the benefits of using them as infrared detectors.

Armchair graphene nanoribbons (AGNRs) are often classified into three families, namely 3*m*, 3*m* + 1, and 3*m* + 2 (*m* is a positive integer). This form of classification is based on the relation between the magnitude of the energy gap and the width of the AGNRs. The quantum confinement effect alters the bandgap energy in these nanostructures, which decreases with the increase of AGNR width (within each group). A comparison of the bandgap of the two structures, i.e., 7-AGNR and 8-AGNR with bandgap energies of 1.47 eV and 0.22 eV, respectively, shows that the bandgap depends on the dimer number or width of the nanoribbons. In other words, the addition of only one row of carbon atoms alters the energy gap about by 1.25 eV. We also used different atoms such as nitrogen, fluorine, and hydrogen to passivate the edge atoms, which yielded different results for the gap size. All these challenges make the production of GNR-based semiconductor materials difficult. However, these challenges are somewhat alleviated in GNMs.

Table 1 summarizes the different GNM structures considered in this study along with the calculated hole index (HI), neck width, and DFT gap. The HI is defined by the ratio of the number of removed carbon atoms to the total number of atoms in the GNM supercell. The results indicate that GNMs with the same HI and size of the supercell have the same bandgap energy. The neck width is another factor determining the GNMs properties. The atoms at the edge of the holes in these materials have been passivated with hydrogen atoms. The results for the gap size with nitrogen passivation are almost the same. In practice, preparing holes with the exact planned atomic structure is very challenging due to effect of atomic edge disorder within the holes. Nguyen et al. [31] showed that the edge disorder in GNMs could entirely alter the electronic properties. To model the disorder, they removed the carbon atoms in the edge of holes with a uniform probability. Their results show that the effect of atomic edge disorder can be considerably reduced by means of proper structure design. Efforts have been made to accurately build such structures.

**Table 1:** Calculated energy gaps for GNMs with hydrogen passivation of carbon atoms in proximity to the holes.

GNM structure	HI = *N*_removed_/*N*_total_ (%)	neck width (nm)	DFT bandgap energy (eV)

A4Z6-6	6.25	1.2	0.49
A4Z6-24	25	0.7	0.92
A4A4-6	6.25	1	0.50
A4A4-24	25	0.5	0.95
Z6Z6-6	8.33	0.7	0.70
Z12Z12-24	8.33	1.7	0.35

**Figure 2:**
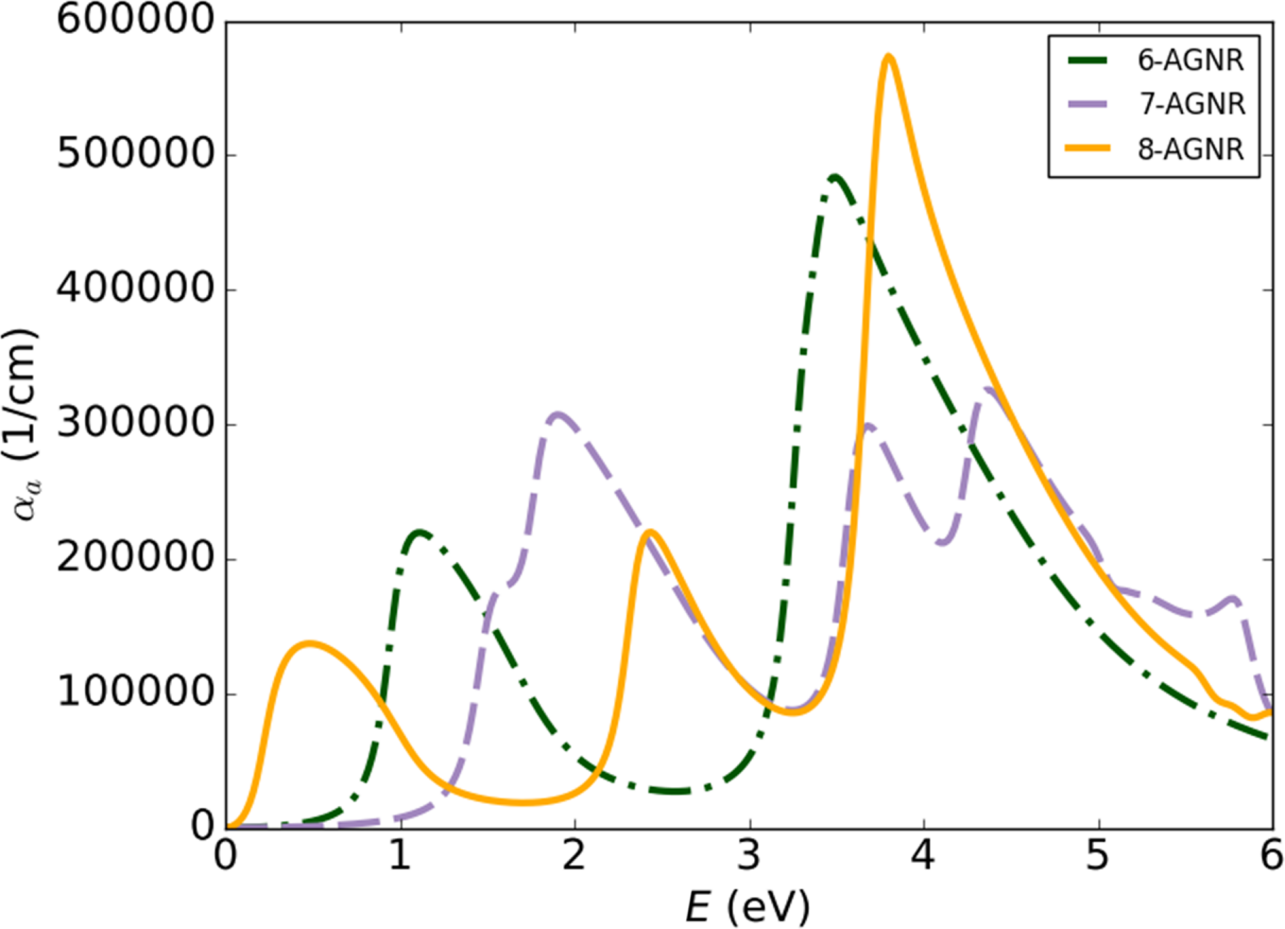
Absorption spectra for different AGNRs. 6-AGNR, 7-AGNR and 8-AGNR.

The formation of bandgaps in these materials is likely to affect their optical properties as well. Figure 2 shows the calculated absorption spectrums of the AGNRs (6-AGNR, 7-AGNR, and 8-AGNR). All of the absorption spectrums appear to have peaks below 2 eV and show a good absorption edge. The peaks in this range are broad and their values are generally around 200,000 cm^−1^.

**Figure 3:**
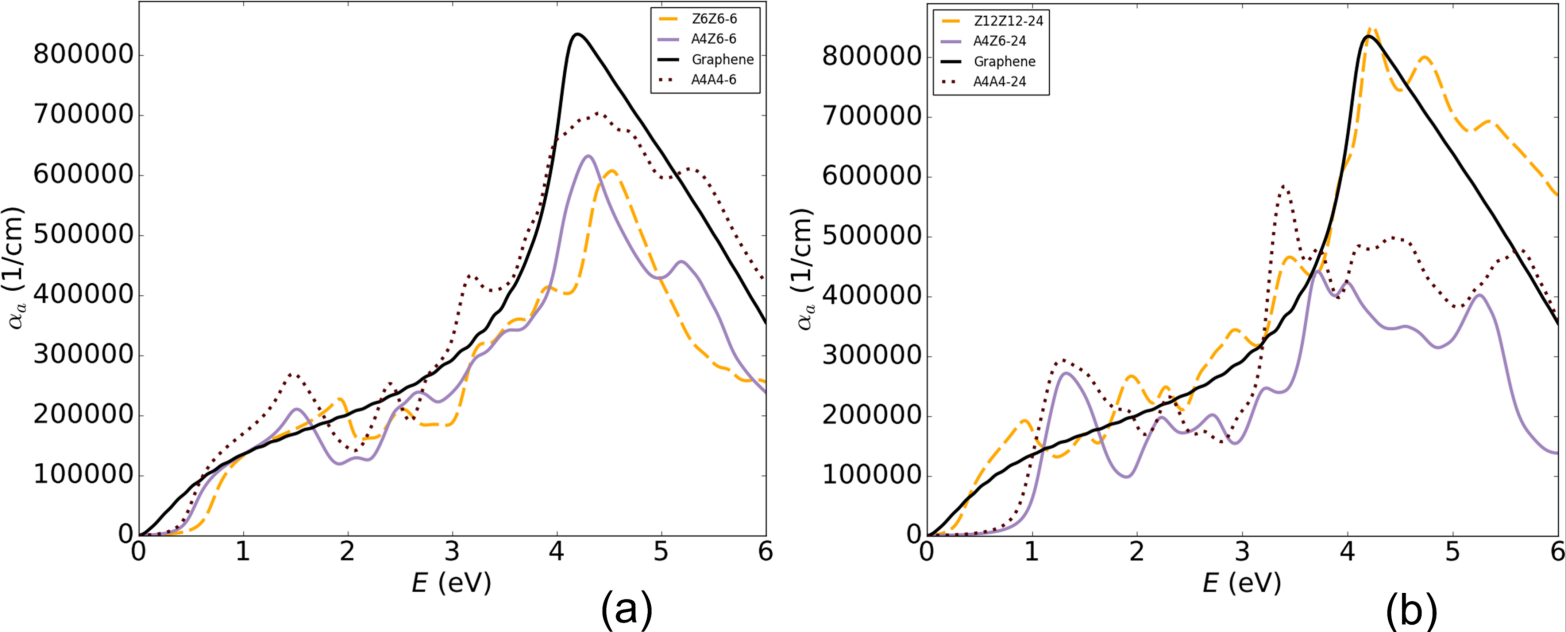
Absorption spectra of different GNM materials. (a) A4A4-6, Z6Z6-6 and A4Z6-6 and (b) A4A4-24, Z12Z12-24 and A4Z6-24.

Figure 3 shows the absorption spectra of different GNM structures. For better comparison, spectra of GNMs with equal hole size are plotted together with the absorption spectrum of graphene. The absorption spectrum of these nanostructures also has a peak in the range of 0 to 2 eV. The maximum value of these peaks is around 300,000 cm^−1^, which is larger than that of GNRs. By comparing the absorption spectra of GNRs and GNMs, it can be concluded that when the ribbon width is increased and, thus, the effect of quantum confinement is reduced, the spectra approach the absorption spectrum of graphene. The same happens when the hole diameter in GNMs is decreased. Also, by increasing the diameter of the holes in the same supercells, i.e., by increasing the HI value (Table 1), the GNM absorption spectrum for a specific neck width becomes similar to the spectrum of the GNR with the analogous width. Peaks at lower energies are also created in structures with higher HI (Figure 3b). The results show that by adjusting the size of the holes in the GNMs, the properties of these materials can be tailored for the fabrication of the photodetectors in the infrared range.

### Device properties

Photodetectors work based on the principle of the internal photoelectric effect, i.e., an incoming photon promotes an electron from the valance band (VB) of a material to the conduction band (CB). The number of electrons produced per incident photon is determined by the quantum efficiency. The electron–hole pairs are then separated due to the bandgap of the material and a bias voltage drives them to the electrodes.

In this section, we use the materials discussed above in a detector structure and obtain the corresponding FOMs. As previously shown (Figure 1), we set up detector configurations based on a confined length of these materials and attach two electrodes to them. Then, we calculate dark current and photocurrent of each structure. The DFTB+NEGF approach is used to calculate the dark current of devices, and for the calculations of the photocurrent, we use first-order perturbation theory in the framework of the Born approximation. In photocurrent calculations, we deal with photon–electron interaction, and according to the polarization of the incoming light, there is a possibility of transferring electrons with different spins (Equation 8). A linear photon polarization in the *X*-direction is assumed for the calculations of the photocurrent.

Table 2 shows some of the main properties (FOMs) of the photodetectors calculated in this study. We obtain these parameters at peak wavelengths of the devices in the optical spectrum. These wavelengths are also reported in Table 2 for each structure.

**Table 2:** Optical properties of different graphene-based structures at 0.3 V bias voltage.

structure	device channel type	detectivity (cm·Hz^1/2^·W^−1^)	responsivity (mA·W^−1^)	peak wavelength (µm)	QE (%)	dark current (μA)

graphene	graphene	3.98 × 10^2^	2.676	1.24	0.268	4.23
6-AGNR	GNR	4.84 × 10^2^	0.894	1.13	0.098	0.79
7-AGNR	GNR	5.51 × 10^2^	0.846	0.65	0.161	0.63
8-AGNR	GNR	9.39 × 10^3^	73.404	2.48	3.67	16.3
A4Z6-6	GNM	7.64 × 10^3^	15.320	0.83	2.289	1.15
A4Z6-24	GNM	8.77 × 10^5^	43.789	0.93	5.838	0.0007

The results show that the performance of GNM-based devices is better than that of other devices at larger wavelengths. For example, the quantum efficiency (QE) of the A4Z6-24 device is 5.8%, which is about 36 times and 1.5 times larger than that of 7-AGNR and 8-AGNR, respectively. The specific detectivity (D*) [7] of the A4Z6-24-based device is about 1000 times larger than that of GNR-based devices and has a responsivity of 43.8 mA·W^−1^, which is about 16 times larger than that of the graphene-based device. The large detectivity of the GNM-based photodetectors is caused by the low dark current and the high QE. It should be noted that this is the first time that the optical properties of GNM-based detectors are computed.

According to the studies, GNM-based transistors have a high on–off (about 100) current ratio similar to that of GNR-based transistors depending on the size of the neck width. For instance, the ratio is higher than 100 for a neck width of approximately 7 nm. GNM-based room-temperature transistors have driving currents that are nearly 100 times higher than those of an individual GNR device [7,32–36]. Next, we computationally evaluate the current transport in devices that are based on semiconducting graphene materials, including GNRs and GNMs, and compare the results. Due to a large number of atoms in the devices that make the calculations costly, we use the DFTB+NEGF approach to facilitate and maintain the accuracy of the transport calculations. To show the accuracy of the DFTB method compared with DFT, we calculated the graphene device current at a bias of 0.3 V in two ways. Using the DFTB+NEGF method a value of 2.58 µA is obtained, while using the DFT+NEGF method the obtained value is 2.29 µA. These two values are very close.

Graphene, GNMs, and GNRs are the three types of materials that we use as channels in these devices. For better comparison, we chose to study the same channel lengths (approximately 5 nm) in all three types of devices. Therefore, the number of periods used along the channel for the GNM devices is three. While applying drain–source voltages from 0 to 1 V we calculate the current transport in the device channel. The effect of the finite bias voltage on the channel potential is calculated using the Poisson equation with the NEGF method.

**Figure 4:**
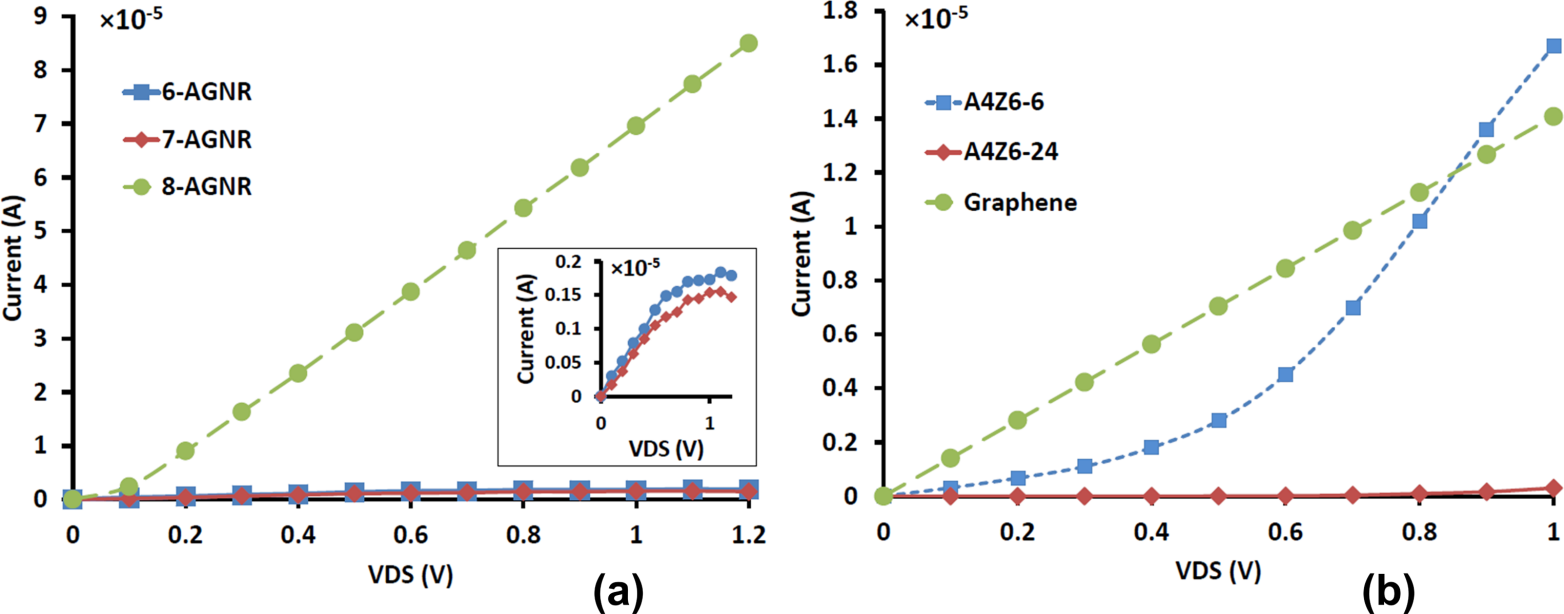
Current–voltage (*I*–*V*) characteristics of (a) different GNR devices and (b) GNM and graphene devices.

Figure 4 shows the current–voltage characteristics of the three types of studied devices. Figure 4a shows that 6-AGNR and 7-AGNR devices have linear *I*–*V* curves over a wide range of the bias voltage. However, the current decreases as the voltage increases further. This indicates a negative differential resistance (NDR) behavior in these devices, which comes from the interaction between the discrete states in the channel region and the narrow density of states in the doped regions [37]. 8-AGNR has a linear curve, and its current at 0.3 V bias is about 20 times higher than that of the other two GNR devices. This is because of the small bandgap of 8-AGNR, which is also visible from the absorption spectrum.

Figure 4b shows the *I*–*V* curves of A4Z6-6 and A4Z6-24 GNMs in comparison with a graphene device. The devices made from GNMs exhibit different transport characteristics than GNR and graphene devices. They have threshold voltage and behave like a diode at positive voltages. These effects are attributed to the formation of a bandgap in the GNMs. A closer look at the *I*–*V* curves in Figure 4 reveals that the driving current of GNM devices is higher at high voltages. As the neck width of the GNMs increases in Figure 4b the current increases. This dependency also occurs in each of the three families of nanoribbons. Therefore, these devices are tunable by varying the neck width. A4Z6-6 exhibits a large current in comparison with A4Z6-24. This is attributed to an increase in the amount of defects in A4Z6-24, i.e., the increasing scattering of carriers decreases the transmission. Another reason is the size of the bandgap. According to our calculations, the bandgap of A4Z6-6 and A4Z6-24 is 0.49 and 0.92 eV, respectively. The Fermi level in A4Z6-6 is closer to the conduction band, which increases the distribution of carriers and the conductivity of this material.

At the end of the paper, we compare our detector to some state-of-the-art detectors. Table 3 summarizes the properties of our device and some of the similar devices previously reported in the literature. This table contains the responsivity of these detectors at given wavelengths. As seen, the GNM photodetector (GNM PD) has a relatively good responsivity and can improve the performance of graphene-based detectors. It should be noted that the GNM PD responsivity is calculated for a single GNM layer, and it can be improved by adding more layers as well as creating various structures based on these materials.

**Table 3:** Responsivity of several photodetectors.

device structure	wavelength (nm)	responsivity (mA·W^−1^)	ref.

GNM PD	930	43.789	this paper
metal–graphene–metal PD	1550	6.1	[5]
graphene p–n junction PD	476	2.7	[12]
waveguide-integrated graphene PD	1550	50	[14]
graphene/Si heterojunction PD	400–900	435	[11]
microcavity-integrated graphene PD	850	21	[9]
MoS_2_/glassy graphene heterostructure PD	532	12.3	[38]
graphene/silicon PD	1550	230	[39]
graphene/germaniun diode PD	1550	52	[40]
black phosphorus PD	1550	5	[41]

## Conclusion

We have simulated GNM-based detectors from top to bottom and obtained their FOMs, which were compared to those of also investigated GNR-based detectors. First, DFT calculations were used to obtain the electronic properties. Later, by using non-equilibrium Green’s functions within a DFTB model and first-order perturbation theory, we have calculated the dark current and photocurrent in these devices.

The results show that GNM-based devices exhibit better optical and electrical properties compared to intrinsic graphene and GNR devices. In terms of transport, GNMs can carry more current than GNRs. In terms of optical properties, they also have larger absorption peaks in addition to peaks in the infrared region. Therefore, detectors based on these materials have higher detectivity, responsivity, and quantum efficiency than pure graphene-based or GNR-based devices. The calculations have shown that the GNM-based detectors have a detectivity that is 1000-fold that of graphene detectors and 100-fold that of GNR devices. In addition, GNMs are tunable materials, meaning that by tuning the diameter and distribution of the defects, their optical and electrical properties can be engineered.
